# Clinical relevance of the *cagA and vacA* s1m1 status and antibiotic resistance in *Helicobacter pylori*: a systematic review and meta-analysis

**DOI:** 10.1186/s12879-022-07546-5

**Published:** 2022-06-25

**Authors:** Mohsen Karbalaei, Amin Talebi Bezmin Abadi, Masoud Keikha

**Affiliations:** 1 Department of Microbiology and Virology, School of Medicine, Jiroft University of Medical Sciences, Jiroft, Iran; 2 Department of Bacteriology, Faculty of Medical Sciences, Tarbiat Modares University, Tehran, Iran; 3 Department of Microbiology and Virology, Faculty of Medicine, Mashhad University of Medical Sciences, Mashhad, Iran

**Keywords:** Antibiotic resistance, *cagA*, *H. pylori*, Treatment, *vacA*

## Abstract

**Background:**

The role of *Helicobacter pylori* (*H. pylori*) virulence factors of such as *vacA* s1m1 and *cagA* in designating clinical outcomes and eradication rate has been deeply challenged in the last decade. The goal of this analysis was to identify the potential relevance between *cagA* and *vacA* genotypes with reported antibiotic resistance observed in clinical *H. pylori* isolates.

**Methods:**

This literature search was conducted in databases such as Clarivate analytics, PubMed, Scopus, EMBASE, DOAJ, and Google Scholar by April 2022, regardless of language restrictions and publication date. Quality of the included studies was assessed by the Newcastle–Ottawa scale. Statistical analysis of retrieved studies was fulfilled using Comprehensive Meta-Analysis software version 2.2. Following quality appraisal of eligible studies, potential association between the status of *cagA* and *vacA* genes with resistance to clarithromycin, metronidazole, amoxicillin, tetracycline, and levofloxacin was measured using odds ratio with 95% confidence interval. We also used sensitivity analyses and meta-regression to eliminate the source of heterogeneity from the overall estimates. Publication bias was assessed using funnel plot, Egger’s test, Begg’s test with the trim and fill procedure to assess the presence and magnitude of publication bias in the included studies.

**Results:**

Our findings suggested that a significant relationship between *cagA* status ‎and increase resistance ‎to metronidazole (OR: 2.69; 95% CI: 1.24–5.83‎‏‎). In subgroup analysis, we ‎found that in the Western ‎population, infection with *cagA*-positive strains could be led to increase in ‎the resistance to ‎metronidazole (OR: 1.59; 95% CI: ‎0.78–3.21‎‏‎), ‎amoxicillin (OR: ‎19.68‎; 95% CI: 2.74–‎‎141.18), ‎and ‎levofloxacin (OR: ‎11.33; 95% CI: ‎1.39–‎‎91.85). After implementation of trim and fill method, the adjusted OR was not significantly differed from original estimates which in turn represented our subgroup analysis was statistically robust. On the other hand, *vacA* ‎genotypes usually ‎reduce the antibiotic resistance of this bacterium, so that *vacA* s1m1 significantly reduces the ‎resistance to ‎metronidazole (OR: 0.41; 95% CI: 0.20–0.86‎‏‎). Surprisingly, resistance of *vacA* s2m2 strains to antibiotics was low, the reason may be due ‎to the non-inflammatory properties of strains containing *vacA* s2m2. The meta-regression and sensitivity analyses successfully reduced the effect of heterogeneity from the overall estimates. In addition, although the pooled OR is reduced after trim and fill adjustment but results do not change the conclusion regarding *vacA* genotypes and antibiotic resistance.

**Conclusions:**

According to our findings, it was clearly demonstrated that *cagA*-positive strains are resistance to metronidazole, especially in Western countries. In Western countries, *vacA* s1m1 increases resistance to amoxicillin and levofloxacin. Based on the present findings, the *vacA* s1m1 genotype significantly increases resistance to metronidazole, while the *vacA* s1m2 decreases resistance to clarithromycin and metronidazole. Resistance to antibiotics in less virulent (*vacA* s2m2) strains is statistically significant lower than others.

## Background

*Helicobacter pylori* (*H. pylori*) is a S-shaped microorganism that colonize in the surface of gastric mucosa of half the world’s population, maybe even more [1]. Long last colonization with this bacterium leads to a chronic progressive gastric inflammation associated with severe gastrointestinal effects [2]. Nowadays, eradication of *H. pylori* is the main therapeutic strategy in management of patients who suffering from different complications including peptic ulcer disease (PUD), gastric cancer (GC), mucosa associated-lymphoid tissue (MALT) lymphoma, and atrophic gastritis [3]. According to the Kyoto Global Consensus Conference, eradication of *H. pylori* infection among the asymptomatic subjects seems an necessity [4]. Nevertheless, the rate of the treatment for *H. pylori* infection is declining annually; the emergence of clarithromycin-resistant strains has been declared a global threat by the World Health Organization (WHO) [5, 6].

The cure rate of *H. pylori* infection could be affected by both microbial (high bacterial load, point mutations, biofilm formation, efflux pumps, and virulence factors), and non-microbial (cytochrome P450 2C19 polymorphism, multidrug resistance transporter-1, pro-inflammatory cytokines polymorphism, smoking, life style, duration of treatment, high gastric acidity, poor patient compliance) factors; all of these factors play a role in the severity of the infection [3, 7, 8]. Vacuolating cytotoxin A (*vacA*) and cytotoxin associated gene A (*cagA*) are considered as the main virulence factors of *H. pylori* [9]. The toxin encoded by the *vacA* gene causes apoptosis, T-cell activation, and persistent infection (through inhibition of immune system), which these changes are lead to severe gastrointestinal outcomes [10]. Full-length sequence analysis of the *vacA* gene showed that this gene has a mosaic structure and is encoded by different subfamilies s1, m1 and m2 alleles, with its own biological activities [11]. The *vacA* s1/m1 genotype possess the highest toxicity property for host cells, while the *vacA* s2/m2 genotype biologically is inactive [12, 13]. CagA is encoded by *cagA* gene; this toxin is highly immunogenic, and upon entering the host cell, it activates kinases through EPIYA motifs in its C-terminal, which in turn disrupt signaling pathways [14].

Studies have shown that this protein induces IL-8 expression, which contributes to the formation of cytokine storms and eventually susceptibility to PUD as well as GC [15]. Both CagA and VacA antigens significantly affect the colonization and pathogenesis of this bacterium, and play a determining role in cure rate of disease [16, 17]. Although chromosomal mutations are considered to be the main mechanism of antibiotic resistance, but, the location of these single nucleotide polymorphisms (SNPs) is not the same in all populations, and therefore, understanding the mechanisms of antibiotic resistance of *H. pylori* is essential for the introduction of rational antibiotic combinations [18]. In recent studies, the eradication results associated with CagA and VacA status are highly inconsistent [19–22]. Interestingly, in meta-analysis by Wang et al. (collecting the data from 26 papers), it was represented that the eradication rate of infection in patients infected with *vacA* s1/*cagA* positive strains was more conducive compared to less virulent strains [8].

In this study, we performed a comprehensive literature search to demonstrate the relationship between *cagA* or *vacA* status and antibiotic resistance in *H. pylori*.

## Methods

### Eligibility of relevant studies

Using international databases such as the Clarivate analytics, PubMed, Scopus, EMBASE, DOAJ, and Google Scholar, related articles to the effect of *cagA* and *vacA* on the antibiotic resistance of *H. pylori* were reviewed, regardless of publication and language restrictions until April 2022. In this regard, we used keywords based on MeSH terms such as “Genotype”, “Antibiotic resistance”, “*Helicobacter pylori*”, “*H. pylori*”, “VacA”, “CagA”, and “Antimicrobial resistance”. The bibliography of articles was reviewed manually to retrieve missing related studies.

### Inclusion and exclusion criteria

Our inclusion criteria were the following: (1) studies on the association between *cagA*/*vacA* status and antibiotic resistance; (2) studies on human subjects; (3) studies based on standard methodology (CLSI); (4) studies without repetitive samples. On the other hand, studies such as case reports, reviews, congress abstracts, duplicates, studies on non *cagA*/*vacA* genes, in vitro studies, as well as studies without clear results were excluded from this study.

### Data extraction

Eligibility of studies was evaluated by the two authors separately, and conflicting of interest was resolved by discussion. The main items were including: first author, country, year of publication, number of *H. pylori* isolates, number of *cagA* + isolates, number of *vacA* s1m1 + isolates, antimicrobial susceptibility tests, and frequency of each genotype (*cagA* and *vacA* s1m1) resistant to clarithromycin, metronidazole, amoxicillin, tetracycline, and levofloxacin (Table 1) [23–63].

**Table 1:** Characteristics of included studies

First author	Country	Year	Number of *H*. *pylori* isolates	Number of *cagA* + *H*. *pylori* isolates	Number of *vacA* s1m1 + *H*. *pylori* isolates	Methods	Number of *H*. *pylori* resistant to clarithromycin		Number of *H*. *pylori* resistant to metronidazole		Number of *H*. *pylori* resistant to amoxicillin		Number of *H*. *pylori* resistant to tetracycline		Number of *H*. *pylori* resistant to levofloxacin		Refs.
							cagA+	vacA s1m1+	cagA+	vacA s1m1+	cagA+	vacA s1m1+	cagA+	vacA s1m1+	cagA+	vacA s1m1+	
Broutet	France	2001	156	84	NR	E-test	8/16	NR	NR	NR	NR	NR	NR	NR	NR	NR	[23]
Solca	Switzerland	2001	71	38	24	NR	6/12	4/12	12/28	9/28	NR	NR	NR	NR	NR	NR	[24]
Toro	Spain	2003	98	63	NR	E-test	1/10	NR	22/38	NR	NR	NR	NR	NR	NR	NR	[25]
Elviss	UK	2004	363	287	149	E-test	1/3	0/3	19/31	6/31	NR	NR	NR	NR	NR	NR	[26]
Elviss	UK	2005	101	81	48	E-test	6/12	5/12	38/53	27/53	NR	NR	NR	NR	NR	NR	[27]
Chihu	Mexico	2005	49	38	NR	E-test	NR	NR	9/11	NR	NR	NR	NR	NR	NR	NR	[28]
Francesco	Italy	2006	62	40	23	E-test	10/15	4/15	NR	NR	NR	NR	NR	NR	NR	NR	[29]
Lai	Taiwan	2006	31	31	NR	E-test	13/53	NR	20/53	NR	NR	NR	NR	NR	NR	NR	[30]
Boyanova	Bulgaria	2009	108	88	NR	Agar dilution method	22/31	NR	35/45	NR	NR	NR	NR	NR	NR	NR	[31]
Taneike	Ireland	2009	103	70	19	E-test	3/17	2/17	10/39	12/39	NR	NR	NR	NR	NR	NR	[32]
Hu	Taiwan	2009	133	127	59	PCR-RFLP	18/18	8/18	NR	NR	NR	NR	NR	NR	NR	NR	[33]
Trespalacios	Columbia	2010	79	NR	NR	E-test	7/14	5/14	21/64	17/64	3/3	2/3	NR	NR	NR	NR	[34]
Agudo	Spain	2010	117	44	NR	E-test	5/34	NR	NR	NR	NR	NR	NR	NR	NR	NR	[35]
Vega	Argentina	2010	299	122	200	Agar dilution	44/83	73/83	73/113	84/113	NR	NR	NR	NR	NR	NR	[36]
Ayala	Mexico	2011	90	NR	NR	E-test	OR: 0.79; 95% CI: 0.11–5.33	OR: 4.76; 95% CI: 0.2–109.7	OR: 0.69; 95% CI: 0.21–2.28	OR: 2.58; 95% CI: 0.59–11.3	NR	NR	NR	NR	NR	NR	[37]
Babab	Japan	2011	35	35	NR	PCR	12/35	NR	NR	NR	NR	NR	NR	NR	NR	NR	[38]
Khan	Pakistan	2012	178	83	NR	Agar dilution	35/64	NR	67/149	NR	20/66	NR	NR	NR	NR	NR	[39]
Yula	Turkey	2013	91	68	NR	Agar dilution	6/7	NR	NR	NR	NR	NR	NR	NR	NR	NR	[40]
Ghotaslou	Iran	2013	99	84	NR	Modified disk diffusion test	16/21	NR	67/97	NR	24/34	NR	NR	NR	NR	NR	[41]
Alfizah	Malaysia	2013	95	NR	49	E-test	NR	NR	NR	15/28	NR	NR	NR	NR	NR	NR	[42]
Rengifo	Colombia	2013	149	NR	NR	Agar dilution	6/7	6/7	NR	NR	7/8	7/8	NR	NR	NR	NR	[43]
Karabiber	Turkey	2014	98	50	NR	Disk-diffusion	4/12	NR	3/6	NR	NR	NR	NR	NR	NR	NR	[44]
Rasheed	Pakistan	2014	46	37	27	E-test	17/22	13/22	28/34	18/34	19/25	14/25	0/2	2/2	NR	NR	[45]
Hussein	Iraq	2015	74	35	42	GenoType HelicoDR kit	3/12	2/12	NR	NR	NR	NR	NR	NR	1/3	2/3	[46]
Boyanova	Bulgaria	2015	84	64	21	E-test	26/26	NR	NR	NR	NR	NR	NR	NR	NR	NR	[47]
Fasciana	Italy	2015	100	48	35	E-test	9/25	12/25	NR	NR	NR	NR	NR	NR	NR	NR	[48]
Liou	Taiwan	2015	1395	597	300	Agar dilution	135/1175	63/578	294/1176	155/577	29/1177	14/579	36/1159	24/564	103/1180	44/581	[49]
Mill´an	Mexico	2016	45	35	36	Disk-diffusion	3/8	3/8	NR	NR	NR	NR	NR	NR	NR	NR	[50]
Miftahussurur	Indonesia	2016	77	73	52	E-test	7/7	6/7	34/36	21/36	NR	NR	NR	NR	22/24	16/24	[51]
Schwetz	Austria	2016	178	100	72	E-test	27/54	21/54	21/35	16/35	NR	NR	NR	NR	17/21	15/21	[52]
Bachir	Algeria	2018	163	97	100	E-test	18/151	18/151	65/151	66/151	NR	NR	NR	NR	NR	NR	[53]
Farzi	Iran	2019	68	57	26	Agar dilution	20/23	10/23	52/56	23/56	18/21	8/21	3/3	1/3	17/19	7/19	[54]
Imkamp	Switzerland	2019	41	19	NR	E-test	14/35	NR	15/30	NR	NR	NR	NR	NR	7/12	NR	[55]
Khani	Iran	2019	61	40	25	E-test	13/48	13/48	NR	NR	NR	NR	NR	NR	NR	NR	[56]
Abdollahi	Iran	2019	63	37	NR	Modified disk diffusion	15/20	NR	22/35	NR	14/17	NR	1/2	NR	NR	NR	[57]
Farzi	Iran	2019	33	29	12	Agar dilution	11/12	4/12	25/33	9/33	9/10	3/10	2/2	1/2	9/9	2/9	[58]
Wang	China	2019	100	87	42	E-test	OR: 2.192; 95% CI: 0.427–11.235	OR: 0.763; 95% CI: 0.287–2.027	OR: 1.509; 95% CI: 0.409–5.561	OR: 0.287; 95% CI: 0.096–0.863	0	OR: 0.434; 95% CI: 0.078–2.420	0	OR: 0.758; 95% CI: 0.215–2.667	OR: 5.133; 95% CI: 1.297–20.319	OR: 0.749; 95% CI: 0.311–1.804	[59]
Glowniak	Poland	2019	62	35	12	E-test	3/4	2/4	6/8	2/8	0	0	0	0	3/4	1/4	[60]
Hamidi	Iran	2020	50	27	8	Agar dilution	7/11	3/11	17/34	3/34	11/16	3/16	5/8	1/8	11/14	3/14	[61]
Haddadi	Iran	2020	128	72	NR	Disk diffusion	4/4	NR	47/52	NR	20/23	NR	5/5	NR	NR	NR	[62]
Okullu	Turkey	2020	33	11	NR	GenoType HelicoDR kit	4/13	NR	NR	NR	NR	NR	NR	NR	NR	NR	[63]
*NR* not reported																	

According to the literature, *vacA* s1m1 is the most virulent genotype of *H. pylori*, nevertheless, in the present meta-analysis, we evaluated the frequency of other *vacA* genotypes in all eligible studies. The distribution of antibiotic resistance of three genotypes *vacA* s1m2, *vacA* s2m1, and *vacA* s2m2 was assessed and their results are shown in Table 2.

**Table 2:** Distribution of antibiotic resistance in vacA genotypes

First author	*vacA* genotypes	Clarithromycin	Metronidazole	Amoxicillin	Tetracycline	Levofloxacin	Refs.
Solca	vacA s1/m2	4/12	8/28	NR	NR	NR	[24]
	vacA s2/m1	1/12	1/28	NR	NR	NR	
	vacA s2/m2	3/12	10/28	NR	NR	NR	
Elviss	vacA s1/m2	1/3	NR	NR	NR	NR	[26]
	vacA s2/m1	NR	NR	NR	NR	NR	
	vacA s2/m2	0/3	2/8	NR	NR	NR	
Elviss	vacA s1/m2	2/3	22/31	NR	NR	NR	[27]
	vacA s2/m1	NR	NR	NR	NR	NR	
	vacA s2/m2	0/3	1/31	NR	NR	NR	
Francesco	vacA s1/m2	6/15	NR	NR	NR	NR	[29]
	vacA s2/m1	NR	NR	NR	NR	NR	
	vacA s2/m2	4/15	NR	NR	NR	NR	
Trespalacios	vacA s1/m2	NR	NR	NR	NR	NR	[34]
	vacA s2/m1	NR	NR	NR	NR	NR	
	vacA s2/m2	2/15	9/15	2/15	NR	NR	
Vega	vacA s1/m2	NR	NR	NR	NR	NR	[36]
	vacA s2/m1	NR	NR	NR	NR	NR	
	vacA s2/m2	10/83	29/113	NR	NR	NR	
Alfizah	vacA s1/m2	NR	12/28	NR	NR	NR	[42]
	vacA s2/m1	NR	NR	NR	NR	NR	
	vacA s2/m2	NR	NR	NR	NR	NR	
Rasheed	vacA s1/m2	7/22	13/34	9/25	0/2	NR	[45]
	vacA s2/m1	NR	NR	NR	NR	NR	
	vacA s2/m2	2/22	3/34	2/25	0/2	NR	
Hussein	vacA s1/m2	2/12	NR	NR	NR	1/3	[46]
	vacA s2/m1	NR	NR	NR	NR	NR	
	vacA s2/m2	3/12	NR	NR	NR	0/3	
Fasciana	vacA s1/m2	4/25	NR	NR	NR	NR	[48]
	vacA s2/m1	NR	NR	NR	NR	NR	
	vacA s2/m2	9/25	NR	NR	NR	NR	
Liou	vacA s1/m2	76/643	162/646	13/645	11/634	62/646	[49]
	vacA s2/m1	0/3	2/3	0/3	0/3	0/3	
	vacA s2/m2	0/5	0/5	0/5	0/5	1/5	
Mill´an	vacA s1/m2	0/8	NR	NR	NR	NR	[50]
	vacA s2/m1	0/8	NR	NR	NR	NR	
	vacA s2/m2	2/8	NR	NR	NR	NR	
Schwetz	vacA s1/m2	14/54	6/35	NR	NR	3/21	[52]
	vacA s2/m1	NR	NR	NR	NR	NR	
	vacA s2/m2	19/54	13/35	NR	NR	3/21	
Bachir	vacA s1/m2	6/38	13/102	NR	NR	NR	[53]
	vacA s2/m1	NR	NR	NR	NR	NR	
	vacA s2/m2	9/38	19/102	NR	NR	NR	
Farzi	vacA s1/m2	11/23	29/56	9/21	2/3	9/19	[54]
	vacA s2/m1	NR	NR	NR	NR	NR	
	vacA s2/m2	2/23	4/56	4/21	0/3	3/19	
Khani	vacA s1/m2	12/48	NR	NR	NR	NR	[56]
	vacA s2/m1	9/48	NR	NR	NR	NR	
	vacA s2/m2	14/48	NR	NR	NR	NR	
Farzi	vacA s1/m2	7/12	14/27	4/10	1/9	5/9	[58]
	vacA s2/m1	NR	NR	NR	NR	NR	
	vacA s2/m2	1/12	4/27	3/10	2/9	0/2	
Hamidi	vacA s1/m2	4/11	14/34	7/16	4/8	5/14	[61]
	vacA s2/m1	NR	NR	NR	NR	NR	
	vacA s2/m2	2/11	4/34	3/16	1/8	1/14	
Glowniak	vacA s1/m2	1/4	3/8	NR	NR	1/4	[60]
	vacA s2/m2	1/4	3/8	NR	NR	2/4	

### Quality assessment

The Newcastle–Ottawa scale (NOS) was used to assess the quality of the included studies. The quality of studies was evaluated based on the items such as selection, comparability, and outcome, so that NOS scores in the range of 1–3, 4–6, and 7–9 were considered low, medium, and high respectively. The quality appraisal process was performed separately by the two authors, and the disagreement was resolved through discussion.

### Statistical analysis

Retrieved studies was analyzed using Comprehensive Meta-Analysis (CMA) software version 2.2 (Biostat, Englewood, NJ, USA). Frequency of *cagA*- and *vacA*-positive strains was measured based on the event rate with 95% confidence interval (95%CI). Finally, the association between the genotypes of these virulence factors and resistance to clarithromycin, metronidazole, amoxicillin, tetracycline, and levofloxacin was calculated using the odds ratio (OR) and corresponding 95% CI. For measuring heterogeneity, we used from two parameters Cochran’s Q statistic and *I*^*2*^ statistic. The fixed-effects model was used when there was no significant heterogeneity (*p* value ≥ 0.10 and *I*^*2*^ ≤ 50%) between the studies [64]; a random-effect model based on the Dersimonian and Laird method was used if significant heterogeneity was identified [65]. Eventually, publication bias was assessed by Egger’s *p* value test, Begg’s *p* value test, and asymmetry of funnel plot [66]. We also used the “trim-fill” method to prove the correction effect on publication bias according to Duval and Tweedie [67, 68]. We performed subgroup analysis based on several items such as ethnicity, study sample size, diagnostic test, and developing/developed status of country. Moreover, the leave-one-out method as sensitivity analyses were performed to estimate the effect of each included study on overall effect [69]. A random effects meta-regression analysis was performed to assess the potential sources of heterogeneity to explore factors that may be associated with between-study variations in *H. pylori* antibiotic resistance.

## Results

### Characteristics of the included studies

**Fig. 1:**
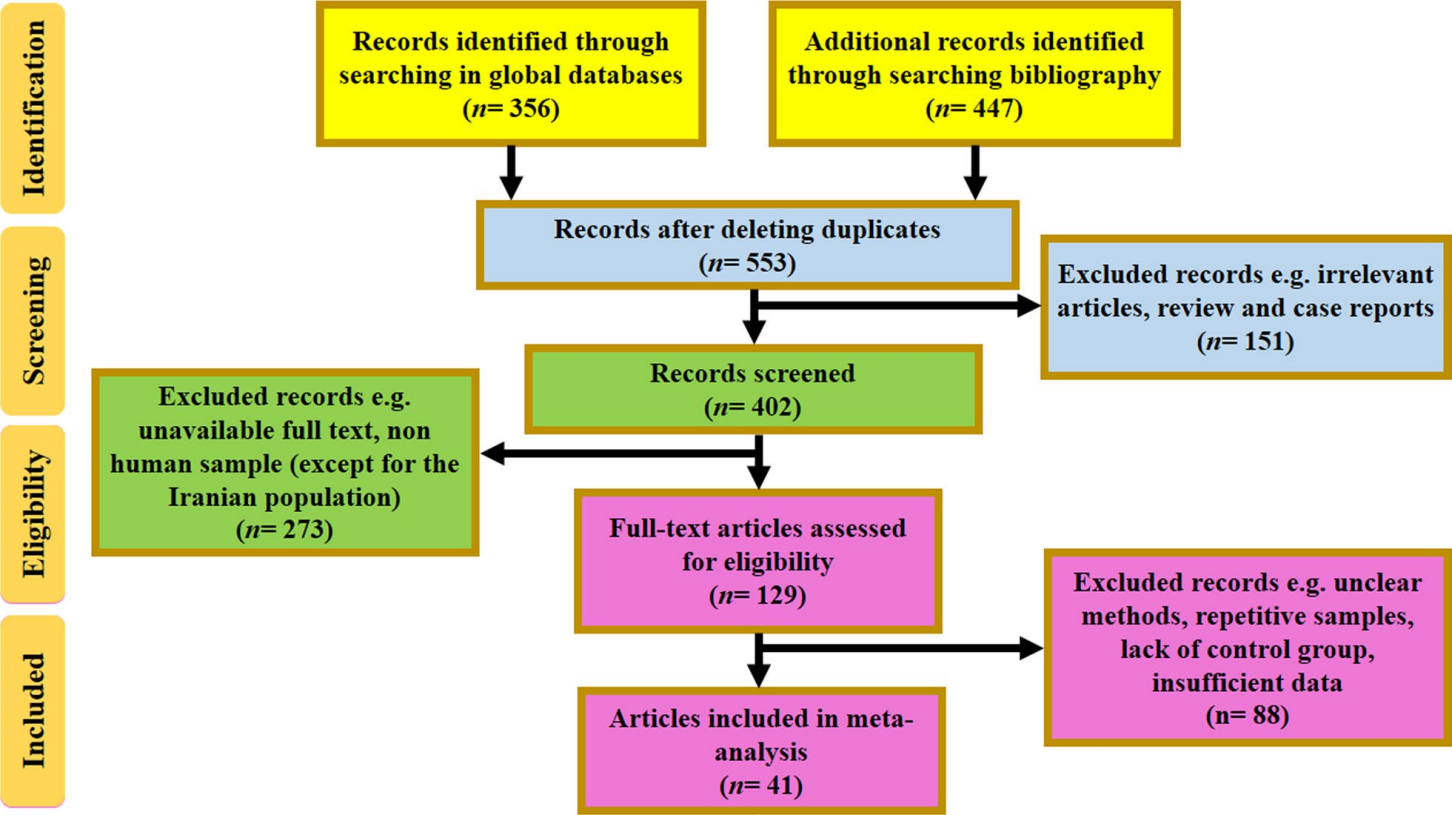
The flowchart of included studies

A systematic literature search was conducted based on PRISMA guideline. In the first stage, 509 articles were selected as potential documents. According to the inclusion criteria 471 articles were deleted and finally 38 eligible articles were entered in the present research (Fig. 1). Of all eligible studies, 38 articles had evaluated the relationship of *cagA* and antibiotic resistance, while 23 articles had assessed the effect of *vacA* genotypes on antibiotic resistance. The NOS results showed that the quality of eligible studies was ranged between 6 and 8. All studies in had been performed in regions such as Asia, Europe, and Latin America during 2001–2020. Standard methods for detecting antibiotic resistance included agar dilution, modified disk-diffusion agar, E-test, PCR-RFLP, GenoType HelicoDR kit. In the present study, 5156 of clinical positive samples were evaluated, and consequently the frequency of infection with *cagA* and *vacA* s1m1 was computed 64.6% (95% CI: 58.4–70.4) and 41.9% (95% CI: 34.3–50.0), respectively.

### The vacA status and antibiotic resistance

**Fig. 2:**
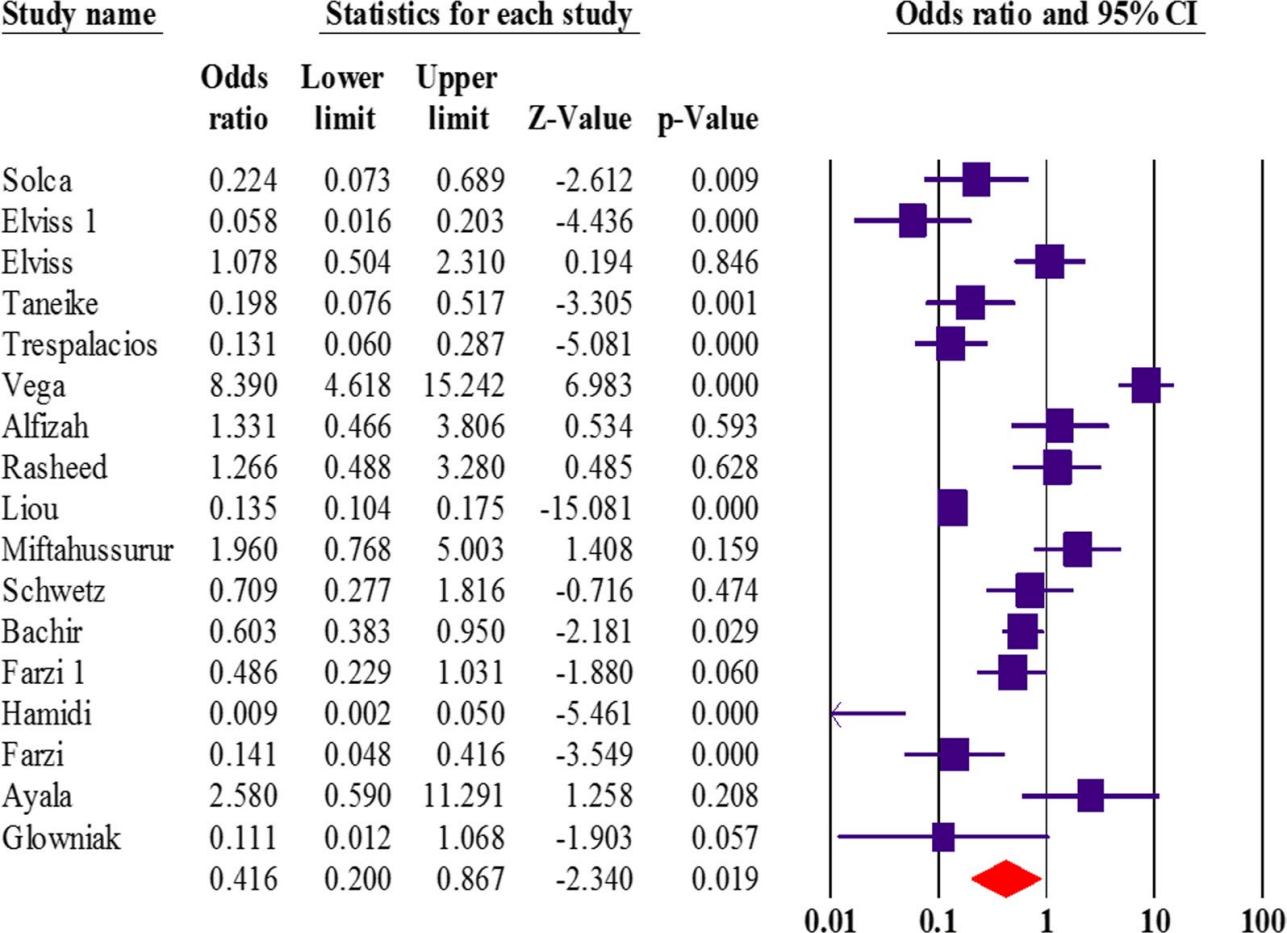
The forest plot associated with *vacA* s1m1 and resistance to metronidazole

**Fig. 3:**
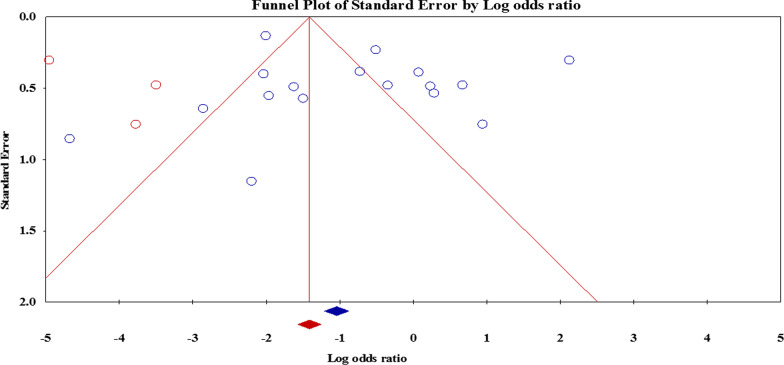
The funnel plot adjusted using a trim and fill method for evaluation of possible link between *vacA* s1m1 and resistance to metronidazole

Overall, 23 articles had appraised the *vacA* genotypes status and resistance to clarithromycin, metronidazole, amoxicillin, tetracycline, and levofloxacin. Interestingly, we found that the *vacA* s1m1 significantly reduced the risk of resistance to metronidazole (OR: 0.41; 95% CI: 0.20–0.86) (Fig. 2). After exclusion 4 studies, the sensitivity analysis was similar (OR: 0.34; 95% CI: 0.29–0.40) without significant heterogeneity rate. Moreover, the results were not significant for other antibiotics (Table 3). Due to the presence of a significant asymmetry in funnel plots, we performed trim and fill method to exclude potential publication bias. Adjusted OR according to the trim-and-fill method was lower than the original estimates but results were similar to the original findings (OR: 0.25; 95% CI: 0.11–0.57); however, a significant difference was not noted between before and after filling the potential missing studies (Fig. 3). Thus, trim and fill method did not change conclusion, indicating that our results were statistically robust regarding potential association between *vacA* s1m1 and resistance to metronidazole.

**Table 3:** Odds ratio (OR) with 95% CI for vacA s1m1 genotype and antibiotic resistance in *H. pylori*

Antibiotic resistance	Random effects model		Heterogeneity		Publication bias	
	OR (95% CI)	*p* value	*p* value	I-squared	Egger’s *p* value	Begg’s *p* value
Clarithromycin	0.40 (0.13–1.22)	0.1	0.01	94.69	0.01	0.79
Metronidazole	0.41 (0.20–0.86)	0.01	0.01	93.54	0.37	0.23
Amoxicillin	0.32 (0.01–5.78)	0.4	0.01	96.70	0.05	0.5
Tetracycline	0.19 (0.007–5.49)	0.3	0.01	94.80	0.1	0.2
Levofloxacin	0.40 (0.03–4.18)	0.4	0.01	97.0	0.04	0.9

The details of overall estimates related to *vacA* s1m1 based on the sample size of the study, diagnostic test, and developing/developed status of country are given in the Table 4.

**Table 4:** The vacA s1m1-positive status and metronidazole resistance

Factors		Random-effects model			Heterogeneity	
		OR	95%CI	*p* value	*p* value	I-squared
Level of country	Developing country	0.30	0.13–0.68	0.01	0.01	86.26
	Developed country	0.55	0.18–1.65	0.01	0.01	93.33
Sample size	≥ 100	1.13	0.84–1.52	0.01	0.31	24.65
	≤ 100	0.28	0.13–0.60	0.01	0.05	64.32
Diagnostic test	E-test	0.64	0.26–1.57	0.3	0.02	58.32
	Agar dilution based	0.25	0.03–1.79	0.17	0.5	32.81
	Disk diffusion based	2.12	0.96–4.67	0.05	0.9	0.00
	Molecular based	1.33	0.46–3.80	0.03	0.9	0.00

In subgroup analysis, the results showed that in an Asian population *vacA* s1m1 significantly increases the resistance of *H. pylori* to metronidazole (OR: 0.37; 95% CI: 0.15–0.90), while in Western countries, *vacA* s1m1 increases resistance to amoxicillin and levofloxacin. (OR: 16.58; 95% CI: 1.77–154.58, and OR: 6.25; 95% CI: 1.63–23.84, respectively). We showed that *vacA* s2m2 decreases resistance to all five antibiotics (clarithromycin, metronidazole, amoxicillin, tetracycline and levofloxacin). On the other hand, *vacA* s1m2 decreases resistance to clarithromycin and metronidazole, while *vacA* s2m1 only decreases resistance to clarithromycin. Details on the relationship between non-*vacA* s1m1 genotypes and antibiotic resistance are summarized in Table 5.

**Table 5:** Odds ratio (OR) with 95% CI for Non-vacA s1m1 genotypes and antibiotic resistance in H. pylori

Non-*vacA* s1m1 genotypes	Antibiotic resistance	Random-effects model		Heterogeneity		Publication bias	
		OR (95% CI )	*p* value	*p* value	I-squared	Egger’s *p* value	Begg’s *p* value
*vacA* s1m2	Clarithromycin	0.13 (0.05–0.16)	0.01	0.01	81.34	0.78	0.88
	Metronidazole	0.32 (0.12–0.81)	0.01	0.01	92.01	0.50	0.20
	Amoxicillin	0.11 (0.003-3.9)	0.2	0.01	97.76	0.02	0.5
	Tetracycline	0.05 (0.001-4.6)	0.2	0.01	95.01	0.05	0.5
	Levofloxacin	0.16 (0.02–1.36)	0.09	0.01	93.48	0.04	0.5
*vacA* s2m1	Clarithromycin	0.03 (0.01–0.09)	0.01	0.01	0.00	0.05	0.7
	Metronidazole	0.07 (0.00-173.5)	0.5	0.01	92.02	NA	NA
	Amoxicillin	0.02 (0.00-1.34)	0.06	0.9	0.00	NA	NA
	Tetracycline	0.02 (0.00-1.34)	0.06	0.9	0.00	NA	NA
	Levofloxacin	0.02 (0.00-1.34)	0.06	0.9	0.00	NA	NA
*vacA* s2m2	Clarithromycin	0.07 (0.03–0.13)	0.01	0.01	55.47	0.02	0.04
	Metronidazole	0.06 (0.02–0.15)	0.01	0.01	84.67	0.52	0.50
	Amoxicillin	0.04 (0.01–0.09)	0.01	0.01	17.61	0.18	0.1
	Tetracycline	0.03 (0.00-0.14)	0.01	0.01	0.00	0.07	0.5
	Levofloxacin	0.03 (0.01–0.12)	0.01	0.01	21.26	0.78	0.5
*NA* not available							

**Fig. 4:**
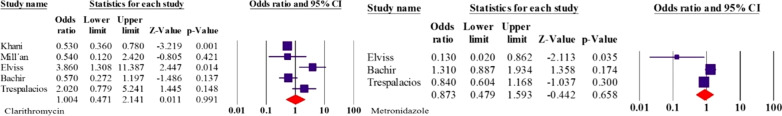
The forest plot associated with *cagA*-*vacA* s1m1 and resistance to clarithromycin and metronidazole

A meta-regression was performed to examine the sources of heterogeneity according to the publication year or NOS score; the results of meta-regression showed that *H. pylori* antibiotic resistance was significantly influenced by publication year (Slope intercept: -0.18; 95% CI: -0.24 to -0.12; SE: 0.029; *p* value: 0.01) or NOS score scale (Slope intercept: -7.30; 95% CI: -8.98 to -5.63; SE: 0.85; *p* value: 0.01). In subgroup analysis, we found no association between the high virulent strains containing *cagA*-*vacA* s1m1 and antibiotic resistance (Fig. 4). In general, it seems that the degree of antibiotic resistance in strains with high pathogenicity is not different from the strains with low virulence. Due to heterogeneity and publication bias, we need further studies with larger sample sizes.

### The cagA status and antibiotic resistance

**Fig. 5:**
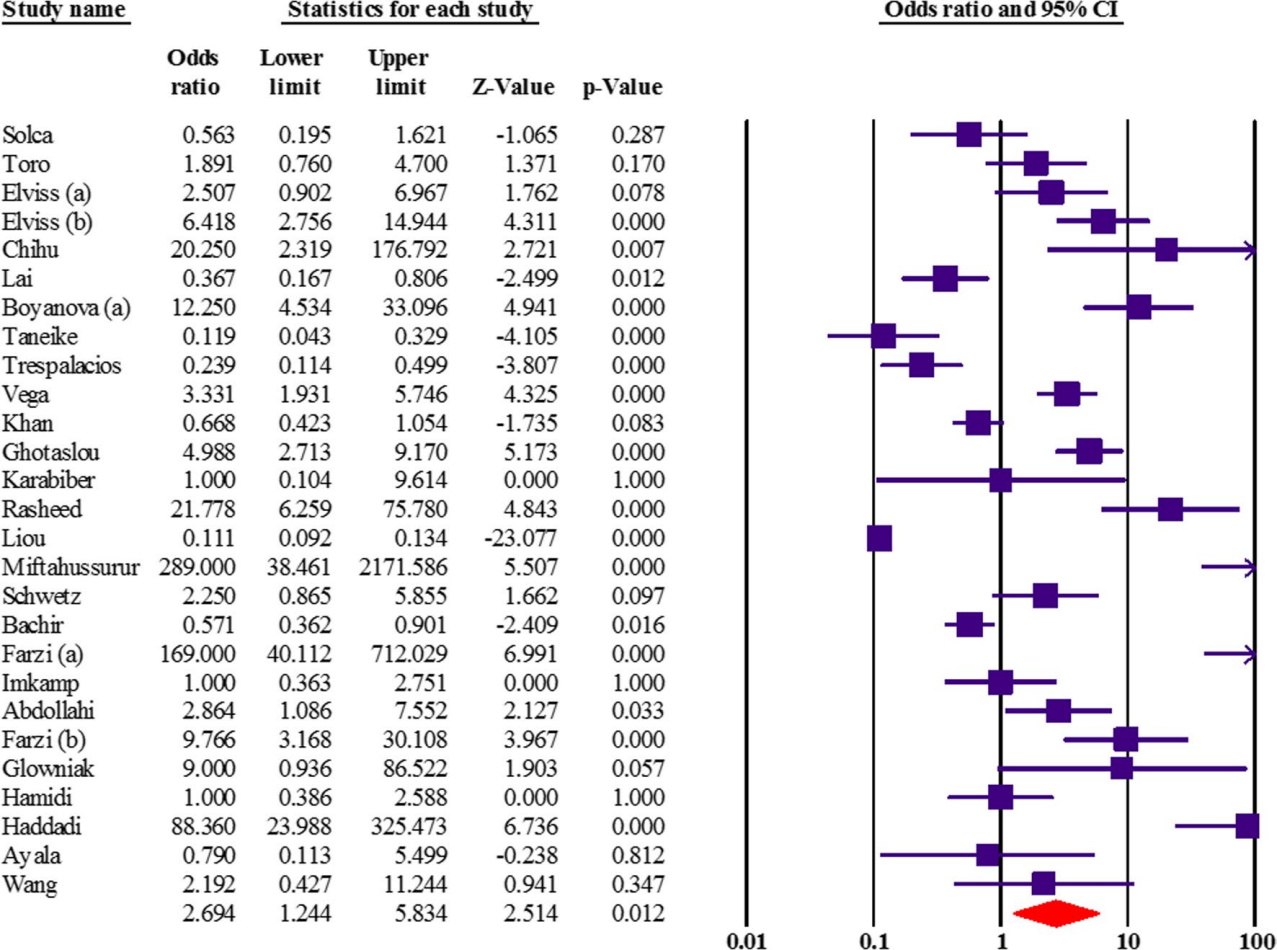
The forest plot associated with *cagA* and resistance to metronidazole

Association between *cagA* status and resistance to clarithromycin, metronidazole, amoxicillin, tetracycline, and levofloxacin had been measured in 40 articles. Based on the current results, it seems that *cagA* significantly increases metronidazole resistance (OR: 2.69; 95% CI: 1.24–5.83; *p* value: 0.01), especially in Western countries (Fig. 5). By discovering the potential sources of heterogeneity, we excluded 3 studies. Sensitivity analysis showed a similar OR: 2.67 (95% CI: 1.20–5.94; *p* value: 0.01). The details of overall estimates related to *cagA* based on the sample size of the study, diagnostic test, and developing/developed status of country are addressed in the Table 6. However, the results of Egger’s regression test and asymmetry of funnel plot showed evidence of publication bias in overall estimates. Thus, we have performed the trim and fill method to adjust for publication bias. The pooled OR did not show the correlation between *cagA* status and antibiotic resistance (OR: 0.29; 95% CI: 0.13–0.64; *p* value: 0.001). Hence, after imputed missing studies by the trim and fill method, the adjusted estimate significantly dropped from OR: 2.69 (95% CI: 1.24–5.83) to OR: 0.29 (95% CI: 0.13–0.64) that revealed there is no relationship between *cagA* status and resistance to metronidazole. The population sample size was low in some included studies that may cause to this significant difference between adjusted OR and original estimates. More extensive research is needed to confirm the present findings.

**Table 6:** The cagA-positive status and metronidazole resistance

Factors		Random-effects model			Heterogeneity	
		OR	95% CI	*p* value	*p* value	I-squared
Level of country	Developing country	2.02	0.84–4.81	0.01	0.01	55.28
	Developed country	3.36	1.14	0.02	0.03	67.45
Sample size	≥ 100	2.02	0.53–7.60	0.01	0.01	98.05
	≤ 100	3.02	1.29–7.043	0.01	0.01	90.97
Diagnostic test	E-test	2.50	1.07–5.83	0.03	0.06	51.69
	Agar dilution based	3.67	0.69–19.47	0.12	0.04	63.97
	Disk diffusion based	1.17	0.41–3.33	0.01	0.93	0.00
	Molecular based	0.23	0.11–0.49	0.01	0.9	0.00

In addition, our findings showed a non-significant association between *cagA* status and resistance to clarithromycin, amoxicillin, tetracycline, and levofloxacin. The results of *cagA* status and resistance to these antibiotics are listed in Table 7. Sensitivity analysis also confirmed the stability of the overall estimates after excluding studies that may cause significant heterogeneity.

**Table 7:** Odds ratio (OR) with 95% CI for cagA genotype and antibiotic resistance in H. pylori

Resistance to	Random-effects model		Heterogeneity		Publication bias	
	OR (95%CI )	*p* value	*p* value	I-squared	Egger’s *p* value	Begg’s *p* value
Clarithromycin	1.61 (0.63–4.11)	0.31	0.01	95.90	0.01	0.62
Metronidazole	2.69 (1.24–5.83)	0.01	0.01	96.42	0.01	0.27
Amoxicillin	5.14 (0.23–114.5)	0.33	0.01	98.46	0.02	0.21
Tetracycline	1.32 (0.01–122.0)	0.95	0.01	95.59	0.01	0.50
Levofloxacin	8.77 (0.24–310.8)	0.21	0.01	98.21	0.01	0.50

A meta-regression was performed to examine the sources of heterogeneity according to the publication year or NOS score; the results of meta-regression showed that publication year (Slope intercept: − 0.150; 95% CI: − 0.20 to − 0.10; SE: 0.025; *p* value: 0.01) or NOS score scale (Slope intercept: − 5.26; 95% CI: − 6.82 to − 3.69; SE: 0.79; *p* value: 0.01) was disrupted the association between *cagA* status and *H. pylori* antibiotic resistance. In the subgroup analysis, our results showed that *cagA* increases resistance to metronidazole, amoxicillin, and levofloxacin only in the Western population (OR: 1.59; 95% CI: ‎0.78–3.21, OR: ‎19.68‎; 95% CI: 2.74–‎‎141.18, and OR: ‎11.33; 95% CI: ‎1.39–91.85‎, respectively), nonetheless, the results associated with the Asian countries were not significant (Table 8). After the trim and fill method, the adjusted OR was slightly lower than original estimates (but not significant difference) which indicates the reliability of the overall estimates.

**Table 8:** Results of subgroup analysis for both Asian and Europe/America (West) populations

Virulence factor/region		Clarithromycin			Metronidazole			Amoxicillin			Tetracycline			Levofloxacin		
Virulence factor	Region	OR	95% CI	*p* value	OR	95% CI	*p* value	OR	95% CI	*p* value	OR	95% CI	*p* value	OR	95% CI	*p* value
*cagA*	Asia	3.12	0.64–15.17	0.1	5.06	1.24–20.12	0.02	3.26	0.10-97.37	0.49	0.73	0.007–83.60	0.9	5.34	0.04–600.0	0.48
	West	0.87	0.31–2.43	0.7	1.59	0.78–3.21	0.1	19.68	2.74-141.18	0.03	NA	NA	NA	11.33	1.39–91.85	0.02
*vacA* s1m1	Asia	0.22	0.06–0.81	0.02	0.37	0.15–0.90	0.03	0.08	0.002–2.91	0.16	0.13	0.004–4.76	0.27	0.22	0.01–0.03	0.27
	West	0.65	0.16–2.52	0.5	0.46	0.13–1.58	0.21	16.58	1.77-154.58	0.01	NA	NA	NA	6.25	1.63–23.84	0.01
*vacA* s1m2	Asia	0.17	0.04–0.71	0.01	0.47	0.14–1.51	0.2	0.11	0.003–3.94	0.22	0.05	0.001–4.66	0.20	0.23	0.01–3.05	0.26
	West	0.10	0.03–0.29	0.01	0.23	0.03–1.41	0.1	NA	NA	NA	NA	NA	NA	0.033	0.006–0.17	0.01
*vacA* s2m1	Asia	0.04	0.01–0.12	0.07	4.00	0.13-119.23	0.40	0.02	0.01–1.34	0.06	0.02	0.01–1.34	0.06	0.02	0.01–1.34	0.06
	West	0.06	0.01–0.06	0.09	0.01	0.00-0.02	0.5	NA	NA	NA	NA	NA	NA	NA	NA	NA
*vacA* s2m2	Asia	0.06	0.02–0.19	0.01	0.012	0.006–0.02	0.01	0.044	0.019–0.12	0.01	0.035	0.008–0.14	0.001	0.02	0.007–0.08	0.01
	West	0.07	0.03–0.15	0.01	0.15	0.05–0.42	0.01	0.024	0.003–0.19	0.001	NA	NA	NA	0.12	0.003–5.75	0.28
*cagA*-*vacA* s1m1	Asia	0.53	0.38–0.75	0.07	1.31	0.88–1.94	0.17	NA	NA	NA	NA	NA	NA	NA	NA	NA
	West	1.87	0.67–4.86	0.23	0.42	0.07–2.45	0.33	NA	NA	NA	NA	NA	NA	NA	NA	NA

*NA* not available

### Publication bias

**Fig. 6:**
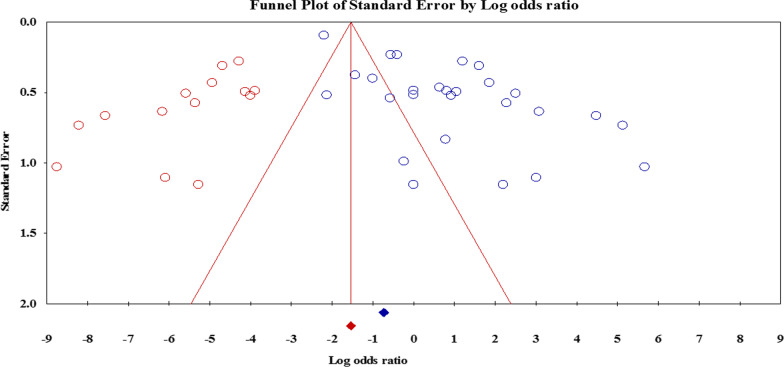
The trimmed and filled funnel plot represent the correlation between the standardized OR and its standard error with pseudo 95% confidence limits regarding possible association between *cagA* status and resistance to metronidazole

The results of Egger’s and Begg’s tests, as well as funnel plot asymmetry showed a significant publication bias; however, when the trim-and-fill method was performed to correct the results, the adjusted OR for *vacA* genotypes was decreased but no significant difference was observed compared to original estimates (Fig. 6). However, the adjusted OR for *cagA* status and resistance to metronidazole was dropped significantly that represents there is no association between *cagA* status and antibiotic resistance.

## Discussion

The *cagA* and *vacA* genes are the most well-known virulence factors of *H. pylori*, and previous studies have demonstrated that infection with *cagA*-*vacA* s1m1 positive strains can increase the risk of severe gastrointestinal disorders [70, 71]. Wang et al. understood that infection with strains carrying both *cagA* and *vacA* products could increase the chance of eradicating *H. pylori* infection, however, the reported heterogeneity was significant [8]. Infection with *cagA*-positive strains can be led to gastric mucosal inflammation, which in turn increases the diffusion of antibiotic (following an increase in blood flow, disruption of mucosal barrier, and inhibition of IL-1β-induced gastric acid secretion) and ultimately high cure rate [72, 73]. Interestingly, *vacA* s1-positive strains reduce the risk of treatment failure due to induce sever gastric inflammation and lower expression of somatostatin [74, 75].

To the best of our knowledge, this is the first meta-analysis study that investigated the potential association between *H. pylori* virulence factor and antibiotic resistance. Based on this analysis, a considerable association exists between the status of *vacA*-*cagA* genes and resistance of *H. pylori* to commonly used antibiotic agents. The results of the present study indicated that *cagA*-positive strains can significantly increase resistance to metronidazole (OR: 2.69; 95% CI: 1.24–5.83; *p* value: 0.01). Although, s1m1 genotype of *vacA* significantly reduces resistance to metronidazole, *vacA* s1m2 reduces resistance to both clarithromycin and metronidazole. Moreover, *vacA* s2m1 decreased resistance to clarithromycin, as well as *vacA* s2m2 decreased resistance to metronidazole, clarithromycin, amoxicillin, tetracycline, and levofloxacin. We showed that *cagA*-positive strains in particular in Western countries increase the risk of resistance to metronidazole, amoxicillin, and ciprofloxacin.

In their study, Chisholm et al. asserted that resistance against metronidazole was not merely due to mutation in the *rdxA* gene, but was influenced by a variety of mechanisms [76]. In a study by Kim et al., they showed that resistance to metronidazole could occur even in the lack of *rdxA* expression or truncated RdxA [77]. Correlation between *cagA* pathogenicity islands (PIA) and resistance to metronidazole first was investigated by Alfizah et al.; they found that strains containing an intact *cag*PAI region were sensitive to metronidazole, while strains possessing partially deleted *cag*PAI regions were resistant to metronidazole [42]. Variations in the 3’ terminal of *cagA* lead to the differentiation of new subclones with unique genetic characteristics, and due to this fact, Rengifo et al. in their study demonstrated that genetic changes in this region cause the formation of antibiotic-resistant subclones [43, 78]. Recent studies show that in patients treated with antibiotics, new subclones of *cagA* are formed due to recombination and quorum sensing, which differ in some features and this phenomenon is effective in antibiotic resistance [79, 80]. We showed that gastric colonization with *cagA*-positive strains, especially in Western countries, can potentially increase the risk of resistance to common antibiotics. In a study conducted by Yue et al., they realized that the prevalence of resistance to metronidazole in strains with Western-type *cagA* 3’ variable region was significantly higher than East Asian-type strains [81, 82]. Today, evidence suggests that CagA protein is involved in processes such as integron acquisition, biofilm formation, and efflux pump function [83–85]. In general, *cagA*-positive strains, especially in the Western population, seem to be considered as diagnostic biomarkers in the phenomenon of antibiotic resistance. Recently, Ayibatari et al. revealed that patients carrying Western-type *cagA* had higher rates of gastritis than East Asian-type *cagA* [86].

Our results showed that *vacA* s2m2 genotype was associated with a significant decrease in resistance to antibiotics. Strains containing *vacA* s2m2 genotype are not able to produce VacA cytotoxic antigen [87]. Krzyżek et al. observed that the change to coccoid form in *vacA* s1m1 strains was significantly higher than *vacA* s2m2 strains [88]. Studies show that *vacA* s2m2 strains have higher nutritional requirements and are also less compatible with antibiotics, so they are more sensitive to antibiotics [89–91]. Though, our results suggested that there is no meaningful association between *cagA*/*vacA* s1m1 double positive *H. pylori* infection and antibiotic resistance. The biofilm formation capacity of *vacA* s1m1 genotype is higher than other genotypes, which in turn is an effective strategy in antibiotic resistance [92, 93]. Our results (as several cross-sectional studies) showed that the s1m1 and s1m2 genotypes reduce the risk of resistance to metronidazole and clarithromycin [59, 94–96]. Strains containing s1 or m1 are strong immunogens to stimulate the immune system and gastritis, so antibiotic delivery in the stomach lumen increases due to increased blood flow [39]. Nevertheless, the effect of other virulence factors may be ignored, for example Brennan et al. showed that the incidence of infection with s1m1/s1m2 strains was higher in treatment-naïve patients than in those previously treated [91].

Overall, our statistical analysis showed that metronidazole resistance was significantly high in *cagA*-positive *H. pylori* strains. As well as, less virulent *vacA* s2m2 genotype was sensitive to all antibiotics. Our study had several limitation including: (1) small ample size; (2) study only on adult population; (3) high heterogeneity among the included studies; (4) imbalanced geographical distribution; (5) inaccessibility to raw data to assess bacterial density and other factors in *cag* PAI; (6) publication bias. However, we performed meta-regression and sensitivity analyses to diminish the effects of heterogeneity on the reliability of the pooled estimates. Meta-regression and sensitivity analyses assisted us exclude the impact of some positive data on the overall estimates. Moreover, we used random-effects models to establish associations among the moderate variables with high heterogeneity. Therefore, it is appropriate to present evidence, but the findings should be interpreted with more caution. In the current meta-analysis, publication bias considerably changed the association between *cagA* status and resistance ‎to metronidazole according to the trim-and-fill method. Meanwhile, adjusted OR for *vacA* genotype and antibiotic resistance after implementation of the trim and fill producer revealed that results were slightly lower without significant difference with overall estimates.

## Conclusions

In the current meta-analysis, our findings showed that infection with *cagA*-positive strains of *H. pylori* significantly increases the risk of metronidazole resistance in Western countries. In addition, *vacA* s1m1 increases resistance to amoxicillin and levofloxacin in Western countries. According to our findings, the *vacA* s1m1 significantly increases resistance to metronidazole, while the *vacA* s1m2 decreases resistance to clarithromycin and metronidazole. Additionally, antibiotic resistance to clarithromycin, metronidazole, amoxicillin, tetracycline, and levofloxacin in less virulent *H. pylori* strains (carrying *vacA*s2m2 genotype) is significantly lower than others. We also performed the trim and fill method to exclude the potential bias from the overall estimates. Although, the adjusted OR was slightly lower than original estimates but this difference was not significant.

## Data Availability

All data generated or analyzed during this study are included in this published article.

## Abbreviations

*H.** pylori*: *Helicobacter pylori*
PU: Peptic ulcer
MALT: Gastric mucosa associated-lymphoid tissue
GC: Gastric cancer
WHO: World Health Organization
*vacA*: Vacuolating cytotoxin A
*cagA*: Cytotoxin associated gene A
PUD: Peptic ulcer disease
SNPs: Single nucleotide polymorphisms
MOS: Newcastle–Ottawa scale
CMA: Comprehensive Meta-Analysis
CI: Confidence interval
OR: Odds ratio

## References

[1] Hooi JK, Lai WY, Ng WK, Suen MM, Underwood FE, Tanyingoh D, Malfertheiner P, Graham DY, Wong VW, Wu JC (2017). Global prevalence of *Helicobacter pylori* infection: systematic review and meta-analysis. Gastroenterology.

[2] Savoldi A, Carrara E, Graham DY, Conti M, Tacconelli E (2018). Prevalence of antibiotic resistance in *Helicobacter pylori*: a systematic review and meta-analysis in World Health Organization regions. Gastroenterology.

[3] Sugimoto M, Yamaoka Y (2009). Virulence factor genotypes of *Helicobacter pylori* affect cure rates of eradication therapy. Arch Immunol Ther Exp.

[4] Sugano K, Tack J, Kuipers EJ, Graham DY, El-Omar EM, Miura S, Haruma K, Asaka M, Uemura N, Malfertheiner P (2015). Kyoto global consensus report on *Helicobacter pylori* gastritis. Gut.

[5] Keikha M, Askari P, Ghazvini K, Karbalaei M (2021). Levofloxacin-based therapy as an efficient alternative for eradicating Helicobacter pylori infection in Iran: a systematic review and meta-analysis. J Glob Antimicrob Resist.

[6] Organization WH. WHO publishes list of bacteria for which new antibiotics are urgently needed. 2017.

[7] Papastergiou V, Georgopoulos SD, Karatapanis S (2014). Treatment of *Helicobacter pylori* infection: meeting the challenge of antimicrobial resistance. World J Gastroenterol WJG.

[8] Wang D, Li Q, Gong Y, Yuan Y (2017). The association between vacA or cagA status and eradication outcome of *Helicobacter pylori* infection: a meta-analysis. PLoS ONE.

[9] Kim I-J, Blanke SR (2012). Remodeling the host environment: modulation of the gastric epithelium by the *Helicobacter pylori* vacuolating toxin (VacA). Front Cell Infect Microbiol.

[10] Sugimoto M, Zali M, Yamaoka Y (2009). The association of vacA genotypes and *Helicobacter pylori*-related gastroduodenal diseases in the Middle East. Eur J Clin Microbiol Infect Dis.

[11] Rhead JL, Letley DP, Mohammadi M, Hussein N, Mohagheghi MA, Hosseini ME, Atherton JC (2007). A new *Helicobacter pylori* vacuolating cytotoxin determinant, the intermediate region, is associated with gastric cancer. Gastroenterology.

[12] Letley DP, Rhead JL, Twells RJ, Dove B, Atherton JC (2003). Determinants of non-toxicity in the gastric pathogen *Helicobacter pylori*. J Biol Chem.

[13] Keikha M, Ali-Hassanzadeh M, Karbalaei M (2020). Association of *Helicobacter pylori* vacA genotypes and peptic ulcer in Iranian population: a systematic review and meta-analysis. BMC Gastroenterol.

[14] Keikha M, Askari P, Ghazvini K, Karbalaei M (2021). Levofloxacin-based therapy as an efficient alternative for eradicating *Helicobacter pylori* infection in Iran: a systematic review and meta-analysis. J Global Antimicrob Resist.

[15] Yamaoka Y, Kita M, Kodama T, Sawai N, Imanishi J (1996). *Helicobacter pylori* cagA gene and expression of cytokine messenger RNA in gastric mucosa. Gastroenterology.

[16] Sgouras DN, Trang TTH, Yamaoka Y (2015). Pathogenesis of *Helicobacter pylori* infection. Helicobacter.

[17] van Doorn L-J, Schneeberger P, Nouhan N, Plaisier A, Quint W, De Boer W (2000). Importance of *Helicobacter pylori* cagA and vacAstatus for the efficacy of antibiotic treatment. Gut.

[18] Miftahussurur M, Waskito LA, Syam AF, Nusi IA, Siregar G, Richardo M, Bakry AF, Rezkitha YAA, Wibawa IDN, Yamaoka Y (2019). Alternative eradication regimens for *Helicobacter pylori* infection in Indonesian regions with high metronidazole and levofloxacin resistance. Infect Drug Resist.

[19] López-Brea M, Martínez MJ, Domingo D, Sánchez I, Alarcón T (1999). Metronidazole resistance and virulence factors in *Helicobacter pylori* as markers for treatment failure in a paediatric population. FEMS Immunol Med Microbiol.

[20] Niu S, Yang F. Relationship between lansoprasoi triple therapy effect and alleles of vacuolating cytotoxin genotype in patients with gastric ulcer. Chinese J Pract Med. 2014; 2(41).

[21] De Magalhaes AN, Carvalhaes A, Natan-Eisig J, Paraiso-Ferraz J, Trevisan M, Zaterka S (2005). CagA status and *Helicobacter pylori* eradication among dyspeptic patients. Gastroenterol Hepatol.

[22] Baryshnikova N, Uspenskiy Y, Suvorov A, Suvorova M (2012). Efficacy of eradication in patients infected with caga (+) and caga (−) strains of *Helicobacter pylori*. Helicobacter.

[23] Broutet N, Marais A, Lamouliatte H, de Mascarel A, Samoyeau R, Salamon R, Mégraud F (2001). cagA Status and eradication treatment outcome of anti-*Helicobacter pylori* triple therapies in patients with nonulcer dyspepsia. J Clin Microbiol.

[24] Solca NM, Bernasconi MV, Valsangiacomo C, Van Doorn L-J, Piffaretti J-C (2001). Population genetics of *Helicobacter pylori* in the southern part of Switzerland analysed by sequencing of four housekeeping genes (atpD, glnA, scoB and recA), and by vacA, cagA, iceA and IS605 genotypingThe GenBank accession numbers for the sequences reported in this paper are AY004351–AY004662. Microbiology.

[25] Toro C, García-Samaniego J, Alarcón T, Baquero M (2003). Relación entre detección de anticuerpos anti-CagA, sensibilidad antibiótica y úlcera péptica en pacientes con infección por *Helicobacter pylori*. Enferm Infect Microbiol Clin.

[26] Elviss NC, Owen RJ, Xerry J, Walker AM, Davies K (2004). *Helicobacter pylori* antibiotic resistance patterns and genotypes in adult dyspeptic patients from a regional population in North Wales. J Antimicrob Chemother.

[27] Elviss NC, Owen RJ, Breathnach A, Palmer C, Shetty N (2005). *Helicobacter pylori* antibiotic-resistance patterns and risk factors in adult dyspeptic patients from ethnically diverse populations in central and south London during 2000. J Med Microbiol.

[28] Chihu L, Ayala G, Mohar A, Hernández A, Herrera-Goepfert R, Fierros G, Gonzalez-Marquez H, Silva J (2005). Antimicrobial resistance and characterization of *Helicobacter pylori* strains isolated from Mexican adults with clinical outcome. J Chemother.

[29] Francesco VD, Margiotta M, Zullo A, Hassan C, Valle ND, Burattini O, D'Angel R, Stoppino G, Cea U, Giorgio F (2006). Claritromycin resistance and *Helicobacter pylori* genotypes in Italy. J Microbiol.

[30] Lai C-H, Kuo C-H, Chen P-Y, Poon S-K, Chang C-S, Wang W-C (2006). Association of antibiotic resistance and higher internalization activity in resistant *Helicobacter pylori* isolates. J Antimicrob Chemother.

[31] Boyanova L, Markovska R, Yordanov D, Marina M, Ivanova K, Panayotov S, Gergova G, Mitov I (2009). High prevalence of virulent *Helicobacter pylori* strains in symptomatic Bulgarian patients. Diagn Microbiol Infect Dis.

[32] Taneike I, Nami A, O’connor A, Fitzgerald N, Murphy P, Qasim A, O’Connor H, O’Morain C (2009). Analysis of drug resistance and virulence-factor genotype of Irish *Helicobacter pylori* strains: is there any relationship between resistance to metronidazole and cagA status?. Alimentary Pharmacol Therapeut.

[33] Hu C-T, Chiou P-Y, Wu C-C, Tseng Y-H, Chang Y-J, Lin N-T (2009). Analysis of resistance to clarithromycin and virulence markers in *Helicobacter pylori* clinical isolates from Eastern Taiwan. Tzu Chi Med J.

[34] Trespalacios AA, Regino WO, Reyes MM (2010). Resistencia de *Helicobacter pylori* a metronidazol, claritromicina y amoxicilina en pacientes colombianos. Rev Colomb Gastroenterol.

[35] Agudo S, Pérez-Pérez G, Alarcón T, López-Brea M (2010). High prevalence of clarithromycin-resistant *Helicobacter pylori* strains and risk factors associated with resistance in Madrid, Spain. J Clin Microbiol.

[36] Vega AE, Cortiñas TI, Puig ON, Silva HJ (2010). Molecular characterization and susceptibility testing of *Helicobacter pylori* strains isolated in western Argentina. Int J Infect Dis.

[37] Ayala G, Galván-Portillo M, Chihu L, Fierros G, Sánchez A, Carrillo B, Román A, López-Carrillo L, Silva-Sanchez J, Study Group J (2011). Resistance to antibiotics and characterization of *Helicobacter pylori* strains isolated from antrum and body from adults in Mexico. Microb Drug Resist.

[38] Baba S, Oishi Y, Watanabe Y, Oikawa R, Morita R, Yoshida Y, Hiraishi T, Maehata T, Nagase Y, Fukuda Y (2011). Gastric wash-based molecular testing for antibiotic resistance in *Helicobacter pylori*. Digestion.

[39] Khan A, Farooqui A, Manzoor H, Akhtar SS, Quraishy MS, Kazmi SU (2012). Antibiotic resistance and cagA gene correlation: a looming crisis of *Helicobacter pylori*. World J Gastroenterol WJG.

[40] Yula E, Nagiyev T, Kaya ÖA, İnci M, Çelik MM, Köksal F (2013). Detection of primary clarithromycin resistance of *Helicobacter pylori* and association between cagA+ status and clinical outcome. Folia Microbiol.

[41] Ghotaslou R, Milani M, Akhi MT, Hejazi MS, Nahaei MR, Hasani A, Sharifi Y. Relationship between drug resistance and cagA Gene in *Helicobacter pylori*. Jundishapur J Microbiol. 2013, 6(10).

[42] Alfizah H, Rukman AH, Norazah A, Hamizah R, Ramelah M (2013). Ethnicity association of *Helicobacter pylori* virulence genotype and metronidazole susceptibility. World J Gastroenterol WJG.

[43] Bustamante-Rengifo JA, Matta AJ, Pazos A, Bravo LE (2013). In vitro effect of amoxicillin and clarithromycin on the 3’region of cagA gene in *Helicobacter pylori* isolates. World J Gastroenterol WJG.

[44] Karabiber H, Selimoglu MA, Otlu B, Yildirim O, Ozer A (2014). Virulence factors and antibiotic resistance in children with *Helicobacter pylori* gastritis. J Pediatr Gastroenterol Nutr.

[45] Rasheed F, Campbell BJ, Alfizah H, Varro A, Zahra R, Yamaoka Y, Pritchard DM (2014). Analysis of clinical isolates of *Helicobacter pylori* in Pakistan reveals high degrees of pathogenicity and high frequencies of antibiotic resistance. Helicobacter.

[46] Hussein N, Tunjel I, Majed H, Yousif S, Aswad S, Assafi M (2015). Duodenal ulcer promoting gene 1 (dupA1) is associated with A2147G clarithromycin-resistance mutation but not interleukin-8 secretion from gastric mucosa in Iraqi patients. N Microb N Infect.

[47] Boyanova L, Markovska R, Yordanov D, Gergova G, Mitov I (2016). Clarithromycin resistance mutations in *Helicobacter pylori* in association with virulence factors and antibiotic susceptibility of the strains. Microb Drug Resist.

[48] Fasciana T, Calà C, Bonura C, Di Carlo E, Matranga D, Scarpulla G, Manganaro M, Camilleri S, Giammanco A (2015). Resistance to clarithromycin and genotypes in *Helicobacter pylori* strains isolated in Sicily. J Med Microbiol.

[49] Liou J-M, Chang C-Y, Chen M-J, Chen C-C, Fang Y-J, Lee J-Y, Wu J-Y, Luo J-C, Liou T-C, Chang W-H (2015). The primary resistance of *Helicobacter pylori* in Taiwan after the national policy to restrict antibiotic consumption and its relation to virulence factors—a nationwide study. PLoS ONE.

[50] Alarcón-Millán J, Fernández-Tilapa G, Cortés-Malagón EM, Castañón-Sánchez CA, De Sampedro-Reyes J, Cruz-del Carmen I, Betancourt-Linares R, Román-Román A (2016). Clarithromycin resistance and prevalence of *Helicobacter pylori* virulent genotypes in patients from Southern México with chronic gastritis. Infect Genet Evol.

[51] Miftahussurur M, Syam AF, Nusi IA, Makmun D, Waskito LA, Zein LH, Akil F, Uwan WB, Simanjuntak D, Wibawa IDN (2016). Surveillance of *Helicobacter pylori* antibiotic susceptibility in Indonesia: different resistance types among regions and with novel genetic mutations. PLoS ONE.

[52] Zollner-Schwetz I, Leitner E, Plieschnegger W, Semlitsch G, Stepan V, Reiter L, Reicht G, Mörth E, Pavek J, Parsché P (2016). Primary resistance of *Helicobacter pylori* is still low in Southern Austria. Int J Med Microbiol.

[53] Bachir M, Allem R, Tifrit A, Medjekane M, Drici A-M, Diaf M, Douidi KT (2018). Primary antibiotic resistance and its relationship with cagA and vacA genes in *Helicobacter pylori* isolates from Algerian patients. Brazilian J Microbiol.

[54] Farzi N, Yadegar A, Sadeghi A, Asadzadeh Aghdaei H, Marian Smith S, Raymond J, Suzuki H, Zali MR (2019). High prevalence of antibiotic resistance in Iranian *Helicobacter pylori* isolates: importance of functional and mutational analysis of resistance genes and virulence genotyping. J Clin Med.

[55] Imkamp F, Lauener FN, Pohl D, Lehours P, Vale FF, Jehanne Q, Zbinden R, Keller PM, Wagner K (2019). Rapid characterization of virulence determinants in *Helicobacter pylori* isolated from non-atrophic gastritis patients by next-generation sequencing. J Clin Med.

[56] Khani S, Abadi ATB, Mobarez AM (2019). Clarithromycin-susceptible but virulent *Helicobacter pylori* strains infecting Iranian patients’ stomachs. Infect Drug Resist.

[57] Abdollahi H, Hashemzadeh M, Khoshnood S, Savari M (2019). Characterization of *Helicobacter pylori* genotypes from Iranian patients with gastric clinical diseases: predominance of vacA s1a and cagA EPIYA-ABC genotypes. Gene Reports.

[58] Farzi N, Yadegar A, Aghdaei HA, Sadeghi A, Zali MR (2019). High prevalence of antibiotic resistance in *Helicobacter pylori* isolates from Iran: importance of functional and mutational analysis of resistance genes and virulence genotyping. BioRxiv.

[59] Wang D, Guo Q, Yuan Y, Gong Y (2019). The antibiotic resistance of *Helicobacter pylori* to five antibiotics and influencing factors in an area of China with a high risk of gastric cancer. BMC Microbiol.

[60] Korona-Glowniak I, Cichoz-Lach H, Siwiec R, Andrzejczuk S, Glowniak A, Matras P, Malm A (2019). Antibiotic resistance and genotypes of *Helicobacter pylori* strains in patients with gastroduodenal disease in Southeast Poland. J Clin Med.

[61] Hamidi S, Badmasti F, Sadeghpour Heravi F, Safapoor MH, Mohammad Ali Tabrizi A, Ghorbani M, Azizi O. Antibiotic resistance and clonal relatedness of *Helicobacter pylori* strains isolated from stomach biopsy specimens in northeast of Iran. Helicobacter. 2020;25(2). 10.1111/hel.12684 32074664

[62] Haddadi M-H, Negahdari B, Asadolahi R, Bazargani A (2020). *Helicobacter pylori* antibiotic resistance and correlation with cagA motifs and homB gene. Postgrad Med.

[63] Oktem-Okullu S, Cekic-Kipritci Z, Kilic E, Seymen N, Mansur-Ozen N, Sezerman U, Gurol Y (2020). Analysis of correlation between the seven important *Helicobacter pylori* (*H. pylori*) virulence factors and drug resistance in patients with gastritis. Gastroenterol Res Pract.

[64] Higgins JP, Thompson SG (2002). Quantifying heterogeneity in a meta-analysis. Stat Med.

[65] DerSimonian R, Laird N (1986). Meta-analysis in clinical trials. Contr Clin Trials.

[66] Karbalaei M, Keikha M (2020). Potential association between the hopQ alleles of *Helicobacter pylori* and gastrointestinal diseases: a systematic review and meta-analysis. Meta Gene.

[67] Duval S, Tweedie R (2000). Trim and fill: a simple funnel-plot-based method of testing and adjusting for publication bias in meta-analysis. Biometrics.

[68] Rothstein HR, Sutton AJ, Borenstein M (2006). Publication bias in meta-analysis: prevention, assessment and adjustments.

[69] Shiota S, Matsunari O, Watada M, Hanada K, Yamaoka Y (2010). Systematic review and meta-analysis: the relationship between the *Helicobacter pylori* dupA gene and clinical outcomes. Gut Pathogens.

[70] Sahara S, Sugimoto M, Vilaichone R-K, Mahachai V, Miyajima H, Furuta T, Yamaoka Y (2012). Role of *Helicobacter pylori* cagA EPIYA motif and vacA genotypes for the development of gastrointestinal diseases in Southeast Asian countries: a meta-analysis. BMC Infect Dis.

[71] Matos JI, de Sousa HA, Marcos-Pinto R, Dinis-Ribeiro M (2013). *Helicobacter pylori* CagA and VacA genotypes and gastric phenotype: a meta-analysis. Eur J Gastroenterol Hepatol.

[72] Maeda S, Yoshida H, Ikenoue T, Ogura K, Kanai F, Kato N, Shiratori Y, Omata M (1999). Structure of cag pathogenicity island in Japanese *Helicobacter pylori*isolates. Gut.

[73] Furuta T, Shirai N, Takashima M, Xiao F, Sugimura H (2002). Effect of genotypic differences in interleukin-1 beta on gastric acid secretion in Japanese patients infected with *Helicobacter pylori*. Am J Med.

[74] Zhao JJ, Wang JB, Yang L, Li Y (2007). Influence of *Helicobacter pylori* genotype on triple eradication therapy. J Gastroenterol Hepatol.

[75] Atherton JC, Tham KT, Peek RM, Cover TL, Blaser MJ (1996). Density of *Helicobacter pylori* infection in vivo as assessed by quantitative culture and histology. J Infect Dis.

[76] Chisholm SA, Owen RJ (2003). Mutations in *Helicobacter pylori* rdxA gene sequences may not contribute to metronidazole resistance. J Antimicrob Chemother.

[77] Kim SY, Joo YM, Lee HS, Chung I-S, Yoo Y-J, Merrell DS, Cha J-H (2009). Genetic analysis of *Helicobacter pylori* clinical isolates suggests resistance to metronidazole can occur without the loss of functional rdxA. J Antibiot.

[78] Vasu K, Nagaraja V (2013). Diverse functions of restriction-modification systems in addition to cellular defense. Microbiol Mol Biol Rev.

[79] Mera R, Fontham ET, Bravo LE, Bravo JC, Piazuelo MB, Camargo MC, Correa P (2005). Long term follow up of patients treated for *Helicobacter pylori* infection. Gut.

[80] Argent RH, Thomas RJ, Aviles-Jimenez F, Letley DP, Limb MC, El-Omar EM, Atherton JC (2008). Toxigenic Helicobacter pylori infection precedes gastric hypochlorhydria in cancer relatives, and *H. pylori* virulence evolves in these families. Clin Cancer Res.

[81] Jin Yong Y, Jing Y, Ming Yi W, Xiao Zhong G. CagA status and genetic characterization of metronidazole resistant strains of *H. pylori* from a region at high risk of gastric cancer. 2014. 10.12669/pjms.304.4840 PMC4121702 25097521

[82] Prazeres Magalhães P, de Magalhães Queiroz DM, Campos Barbosa DV, Aguiar Rocha G, Nogueira Mendes E, Santos A, Valle Corrêa PR, Camargos Rocha AM, Martins Teixeira LC, Affonso de Oliveira C (2002). *Helicobacter pylori* primary resistance to metronidazole and clarithromycin in Brazil. Antimicrob Agents Chemothera.

[83] Kawai M, Furuta Y, Yahara K, Tsuru T, Oshima K, Handa N, Takahashi N, Yoshida M, Azuma T, Hattori M (2011). Evolution in an oncogenic bacterial species with extreme genome plasticity: *Helicobacter pylori* East Asian genomes. BMC Microbiol.

[84] Wong EHJ, Ng CG, Chua EG, Tay ACY, Peters F, Marshall BJ, Ho B, Goh KL, Vadivelu J, Loke MF (2016). Comparative genomics revealed multiple *Helicobacter pylori* genes associated with biofilm formation in vitro. PLoS ONE.

[85] Kyrillos A, Arora G, Murray B, Rosenwald AG (2016). The presence of phage orthologous genes in *Helicobacter pylori* correlates with the presence of the virulence factors CagA and VacA. Helicobacter.

[86] Ayibatari A, Galleh RP, Ogo AC, Anzaku AA, Akyala AI (2021). Prevalence of virulence genes and associated risk factors of *Helicobacter pylori* infection among adults in gastric cancer risk region of North Central, Nigeria. Eur J Clin Biomed Sci.

[87] Correa P, Piazuelo MB (2008). Natural history of *Helicobacter pylori* infection. Digest Liver Dis.

[88] Krzyżek P, Biernat MM, Gościniak G (2019). Intensive formation of coccoid forms as a feature strongly associated with highly pathogenic *Helicobacter pylori* strains. Folia Microbiol.

[89] Yaqub M, Ghezzi P (2015). Adding dimensions to the analysis of the quality of health information of websites returned by Google: cluster analysis identifies patterns of websites according to their classification and the type of intervention described. Front Public Health.

[90] Mendoza-Elizalde S, Arteaga-Resendiz NK, Valencia-Mayoral P, Luna RC, Moreno-Espinosa S, Arenas-Huertero F, Zúñiga G, Velázquez-Guadarrama N (2016). Diversification of the vacAs1m1 and vacAs2m2 Strains of *Helicobacter pylori* in *Meriones unguiculatus*. Front Microbiol.

[91] Brennan DE, Dowd C, O’Morain C, McNamara D, Smith SM (2018). Can bacterial virulence factors predict antibiotic resistant *Helicobacter pylori* infection?. World J Gastroenterol.

[92] Cellini L, Grande R, Di Campli E, Di Bartolomeo S, Di Giulio M, Traini T, Trubiani O (2008). Characterization of an *Helicobacter pylori* environmental strain. J Appl Microbiol.

[93] McClain MS, Cao P, Iwamoto H, Vinion-Dubiel AD, Szabo G, Shao Z, Cover TL (2001). A 12-amino-acid segment, present in type s2 but not type s1 *Helicobacter pylori* VacA proteins, abolishes cytotoxin activity and alters membrane channel formation. J Bacteriol.

[94] Boehnke KF, Valdivieso M, Bussalleu A, Sexton R, Thompson KC, Osorio S, Reyes IN, Crowley JJ, Baker LH, Xi C (2017). Antibiotic resistance among *Helicobacter pylori* clinical isolates in Lima, Peru. Infect Drug Resist.

[95] Feliciano O, Gutierrez O, Valdés L, Fragoso T, Calderin AM, Valdes AE, Llanes R. Prevalence of *Helicobacter pylori* vacA, cagA, and iceA genotypes in Cuban patients with upper gastrointestinal diseases. BioMed Res Int. 2015; 2015. 10.1155/2015/753710 PMC4402555 25945344

[96] Pajavand H, Alvandi A, Mohajeri P, Bakhtyari S, Bashiri H, Kalali B, Gerhard M, Najafi F, Abiri R. High frequency of vacA s1m2 genotypes among *Helicobacter pylori* isolates from patients with gastroduodenal disorders in Kermanshah, Iran. Jundishapur J Microbiol. 2015, 8(11). 10.5812/jjm.25425 PMC4740511 26862378

